# What makes Alpine swift ascend at twilight? Novel geolocators reveal year-round flight behaviour

**DOI:** 10.1007/s00265-017-2438-6

**Published:** 2018-02-26

**Authors:** Christoph M. Meier, Hakan Karaardıç, Raül Aymí, Strahil G. Peev, Erich Bächler, Roger Weber, Willem Witvliet, Felix Liechti

**Affiliations:** 1 Swiss Ornithological Institute, Seerose 1, 6204 Sempach, Switzerland; 2 Elementary Science Education Department, Education Faculty, Alanya Alaaddin Keykubat University, 07400 Alanya, Turkey; 3 Catalan Ornithological Institute, Museu de Ciències Naturals de Barcelona, Pl. Leonardo da Vinci, 4-5, 08019 Barcelona, Spain; 4 Institute of Biodiversity and Ecosystem Research, Bulgarian Academy of Sciences, 2, Gagarin Street, 1113 Sofia, Bulgaria; 5 Bern University of Applied Sciences Engineering and Information Technology, Jlcoweg 1, 3400 Burgdorf, Switzerland; 6 Willem Witvliet, Zuidersloot 16, 1741 Broek op Langedijk, HL Netherlands

**Keywords:** Flight altitude, Bird migration, Annual cycle, Daily activity, Orientation

## Abstract

**Abstract:**

Studying individual flight behaviour throughout the year is indispensable to understand the ecology of a bird species. Recent development in technology allows now to track flight behaviour of small long-distance bird migrants throughout its annual cycle. The specific flight behaviour of twilight ascents in birds has been documented in a few studies, but only during a short period of the year, and never quantified on the individual level. It has been suggested that twilight ascents might be a role in orientation and navigation. Previous studies had reported the behaviour only near the breeding site and during migration. We investigated year-round flight behaviour of 34 individual Alpine swifts (*Apus melba*) of four different populations in relation to twilight ascents. We recorded twilight ascents all around the year and found a twofold higher frequency in ascents during the non-breeding residence phase in Africa compared to all other phases of the year. Dawn ascents were twice as common as dusk ascents and occurred mainly when atmospheric conditions remained stable over a 24-h period. We found no conclusive support that twilight ascents are essential for recalibration of compass cues and landmarks. Data on the wing flapping intensity revealed that high activity at twilight occurred more regularly than the ascents. We therefore conclude that alpine swift generally increase flight activity—also horizontal flight—during the twilight period and we suppose that this increased flight activity, including ascents, might be part of social interactions between individuals.

**Significance statement:**

Year-round flight altitude tracking with a light-weight multi-sensor tag reveals that Alpine swifts ascend several hundred meters high at twilight regularly. The reason for this behaviour remains unclear and the low-light conditions at this time of the day preclude foraging as a possibility. The frequency and altitude of twilight ascents were highest during the non-breeding period, intermediate during migration and low for active breeders during the breeding phase. We discuss our findings in the context of existing hypotheses on twilight ascent and we propose an additional hypothesis which links twilight ascent with social interaction between flock members. Our study highlights how flight behaviour of individuals of a migratory bird species can be studied even during the sparsely documented non-breeding period.

**Electronic supplementary material:**

The online version of this article (10.1007/s00265-017-2438-6) contains supplementary material, which is available to authorized users.

## Introduction

We know very little about how birds use the aerial space (Bowlin et al. 2015). The largest source of information comes from radar studies, where the altitude of migrating birds can be measured as they pass over a ground station (Dokter et al. 2011; Kemp 2012). Tracking devices offer an alternative source of information, but can only be attached to relatively large birds which can carry sufficient battery load, e.g. such as a GPS receiver (e.g., Duriez et al. 2009; Klaassen et al. 2012; Bridge et al. 2013).

As a result, our knowledge about 3D flight behaviour in birds is limited to circumstances when local factors like wind or orographic barriers confine birds to fly on a certain flight altitude (Liechti 2006; Hawkes et al. 2013; Shamoun-Baranes et al. 2017). In contrast, only a few studies have examined flight behaviour on a temporal scale (Klaassen et al. 2011; Tarroux et al. 2016) and we know very little about how flight behaviour might change along a bird’s track, or over different seasons (Shamoun-Baranes et al. 2017, but see, e.g. Liechti et al. 2013; Hedenström et al. 2016). A rare example for vertical flight behaviour is that of twilight ascents during migration. Indeed, several studies have found birds to ascend several hundred meters before returning again to a lower altitude after less than an hour before sunrise and after sunset. The first reports of this phenomenon came from radar ornithologists observing waterfowl flying near the water surface at night before making a sudden ascent at dawn (Myres 1964; Richardson 1978; Diehl et al. 2003). The authors assumed that the behaviour was caused by birds needing to orienteer themselves towards the shore and thus needing the altitude to locate their next stopover site for the day (Bourne 1980). As expected, birds tended to change their direction after the ascent (Diehl et al. 2003). Another explanation could have been that birds were shifting flight altitude in response to a change in wind conditions as convection over land picked up with the first sunlight (Richardson 1978). In a different case, swallows simultaneously ascended at dawn immediately after leaving their night roost most likely as an anti-predator strategy against hunting falcons which had specialised on roost sites (Bijlsma and van den Brink 2005). Very similar ascents were observed in Eleonora’s falcon which climbed at dawn to spot passing-by migrants, as prey from a high altitude (Walter 1979).

However, neither of the afore-mentioned reasons fit as an explanation for symmetrical ascents during dusk and dawn in common swifts (*Apus apus*). The phenomenon has first been reported by Buurma (1987, 2000) and documented in detail by Dokter et al. (2013) using radar. During the breeding season (May–July), thousands of common swifts regularly ascended in the Netherlands above Lake Ijssel for about an hour up to 2.5 km above ground level (AGL) between the civil and nautical twilight (solar elevation − 6° to − 12°). With no apparent relationship between the swifts’ flight altitude and insect abundance, the authors concluded that the swifts did not ascend to forage (Dokter et al. 2013). Instead, the authors suggested the ascents were directly linked with the simultaneously occurring sunrise and sunset and suggested that swifts may gather information for orientation or on weather conditions.

Firstly, at twilight, the polarisation pattern in the sky is at its maximum and reliably revealing the position of the sun even on an overcast sky (Hegedüs et al. 2007). This cue can help birds to recalibrate their compass orientation (Muheim et al. 2006). Also, the bird’s sensor to detect the Earth magnetic field is probably light-dependent (Muheim et al. 2002; Mouritsen and Ritz 2005) and likely to have increased sensitivity at intensities of crepuscular light (Cochran et al. 2004; Muheim et al. 2014). Therefore, we could speculate that birds ascending at twilight might have a maximum set of cues available to recalibrate their sense of orientation. They can oversee an increased number of landmarks and bring them in line with cues for the polarisation pattern and the magnetic field. This might be especially important when moving distances on a great scale within the Earth’s magnetic field (Chernetsov 2016).

Secondly, the buildup of the atmosphere markedly changes between night and day with major implication for flying birds (Shamoun-Baranes et al. 2017). During the day, the lowest layer is mainly governed by convection and vertical air movement causing turbulences (Rohli and Vega 2013). This structure collapses at dawn and the lowest layer becomes dominated by laminar horizontal air flow. This diurnal pattern makes the twilight period an important moment for birds to update their information on stability and current structure of the atmosphere. With an ascent, birds could be able to spot approaching clouds from weather fronts (e.g., an ascent of 300 m can reveal thunderstorm clouds of 10-km height in a distance of 60 km behind the horizon) and they could also compile a temperature profile at different air layers (Cronin et al. 2006; Kemp 2012). This information might be relevant for birds to locate insects—the exclusive food source of swifts (Arn 1960; Collins et al. 2009). In fact, the abundance of insects depends on temperature and precipitation and insects might passively drift through the air (Reynolds et al. 2005; Chapman et al. 2011).

In the case of the common swift, Dokter et al. (2013) observed a correlation of the ascent height with altitude of the 280 K isocline but otherwise found no influence of any other weather variables on ascent height. Thus, they concluded that ascents probably played a role in orientation and that the phenomenon should be further investigated.

Recently, Liechti et al. (2013) tracked free-living Alpine Swifts over the entire season using a novel geolocator with an integrated accelerometer and showed that these birds can stay airborne for more than 200 days. This study provided additional evidence that Alpine swifts are also preforming twilight ascents and possibly could serve as an ideal model system to elucidate where and when birds make twilight ascents during the entire annual cycle. Though the system is not perfect because light-level geolocators lack the accuracy to determine the context of the ascent and hence are insufficient to investigate any kind of relationship between accents and food abundance, predator avoidance and orientation towards local stopover site, respectively. However, by combining geolocators with other sensors, we can gain insight into how general the phenomenon of twilight ascent is, which life history stage these ascents are performed at, at which approximate location and in what weather conditions.

Here, we present data on year-round flight behaviour of individual Alpine swifts based on a novel light-weight tag with sensors allowing to measure light-level, pressure and acceleration. With these data, we can show for each twilight event whether the bird made an ascent and test two specific hypotheses. Firstly, the ‘recalibration hypothesis’—indeed, if twilight ascents are required for recalibrating the orientation sensors at twilight, we would expect them to occur more frequently during migration relative to breeding and non-breeding residence periods, when birds regularly come across new landmarks. Secondly, the ‘atmosphere profiling hypothesis’—indeed, if twilight ascents provide updated information on atmospheric conditions, we would expect them to occur more frequently before and after the passage of weather fronts, when atmospheric conditions are unstable and fluctuate quickly.

## Methods

### Study species

Alpine swifts weigh 90–100 g, are obligatory aerial plankton feeder and spend most of their lifetime, except for the breeding phase, aloft (Arn 1960; Liechti et al. 2013). Their distribution range extends all around the Mediterranean Sea and to the far Middle East, with some breeding colonies north of the Alps, mainly in Switzerland, and one isolated population in South Africa (Cramp 1985). Alpine swifts breed colonially in rock cavities and manmade constructions (Arn 1960; Cramp 1985). The Swiss population breeds is between May and August and it migrates in September–October and March–April (Glutz von Blotzheim et al. 1988; Arn 1960). A first geolocator study observed three birds from Switzerland which flew to a non-breeding range in the Guinean mountain and Togo mountain range in West Africa (Liechti et al. 2013).

### Geolocator tags

In 2014, we equipped a total of 76 alpine swift (Apus melba) with multi-sensory geolocator tags (SOI-GDL3pam) in five different breeding colonies in Baden (47.47° N, 8.31° E, 14 tags); Lenzburg, Switzerland (47.39° N, 8.18° E, 13 tags); on Pırasalı Island, Tukey (36.34° N, 30.53° E, 25 tags); in Tarragona, Spain (41.12° N, 1.25° E, 4 tags) and in Sofia, Bulgaria (42.66° N, 23.34° E, 20 tags). These tags weighted 1.4 g including the harness. They recorded automatically—thus with a blind method—actual values of light intensity and acceleration (vertical axis only, details see Liechti et al. 2013) every 5 min and air pressure and temperature every 30 min. Acceleration was only recoded in vertical direction to save on battery and memory space. From this measure, it was possible to derive the pitch of the axis of the bird and activity. Activity was almost entirely generated by flapping activity, as it was defined as the cumulative change in acceleration in z-direction measured at 10 Hz over a 3.2-s period. Pitch was derived from the component of gravitational acceptation pointing in z-direction and it was calculated as the mean acceleration over the same 3.2-s period. It was discarded from our analysis since it did not provide relevant additional information for this investigation (for details, see Liechti et al. 2013). With all the setting above, the tag was able to record for almost the entire annual cycle (from the end of the breeding season until the start of the consecutive breeding season).

### Tag analysis

Light-level data were used to calculate the approximate position of each bird at each twilight event (sunrise and sunset) using the R-package FLightR (Rakhimberdiev et al. 2015; R Core Team 2016; Rakhimberdiev and Saveliev 2016). FLightR is a hidden Markov model combining a template fit and a simple random walk movement mode for geo-positioning of every sun event (dawn and dusk separately). Unnatural sun events, which in our data often occurred when birds stayed inside the breeding cavity during twilight, had to be discarded prior to the analysis with FLightR. Unnatural sun events were clearly recognisable by an abrupt increase or decrease of the light values between two consecutively measuring intervals and could be identified with a manual inspection from the raw light-level data. The final output of FLightR consists of a spatial map with the probability distribution for the geographical position at each sun event. The map then provides inferences about the uncertainty of the bird’s position in time and space. We will only report the part of the FLightR analysis (see below) which is relevant to test our hypothesis about vertical flight behaviour of the alpine swift. The full details on the route and timing of migration will be published elsewhere. All tags were analysed using the same initial parameter settings for FLightR (see supplementary material).

The recognition of twilight ascents required information on the bird’s flight altitude over the course of the day and the time of the twilight event. The time of the twilight event could either be inferred from light-level data on days with a natural twilight event using the threshold method (Lisovski and Hahn 2012) or it had to be calculated with the astronomical formula on the day when the light sensor had only recorded an unnatural sun event. For the latter, we used the R-function solarpos from the package maptools (Bivand and Lewin-Koh 2015). This function required the approximate position of the bird, which was linearly interpolated between the last and proximate median position provided by the FLightR analysis. We assumed that birds resided at the breeding colony in autumn between the day the tag was mounted and the first day with a natural sun event and in spring after the last day with a natural sun event and the day the tag was removed.

Flight altitude *h* could be calculated from air pressure using the international barometric formula (Riegel 1992),

1 $$ h=\frac{T_0}{\frac{dT}{dh}}\times \left(1-{\left(\frac{p_h}{p_0}\right)}^{\frac{\left(\upkappa -1\right)}{\upkappa}}\right) $$

with the standard assumption *T*_*0*_ = 288.15 K (=15 °C) for the sea level standard temperature, *dT*/*dh* = 0.65 K/100 m for the temperature gradient and *κ* = 1.235 for the heat capacity ratio. *P*_*h*_ was the pressure measured by the tag on the bird and *P*_0_ was the pressure at sea level provided by the NOAA database (see below) for the approximate median position of the bird according to the FLightR output. Because exact positions are not available by light-level geolocation pressure data can only reveal flight altitudes above sea level, but not above ground.

Since pressure was measured on a 30-min interval, we had to approximate the flight altitude at twilight by averaging the two closest measures to the twilight event. The height of a twilight ascent was then defined as the difference between this average altitude and the average flight altitude for the 11 closest hours around the twilight event (6 h before and 6 h after, excluding the hour with twilight itself). Activity measures were used to differentiate between gliding flight and flapping flight behaviour as described in Liechti et al. (2013).

### Hypothesis testing

Our first hypothesis posits that twilight ascents should occur with the highest intensity during the migration phases because the individuals have to update their orientation at every new site. Testing this hypothesis required assigning each twilight ascents to one of the three discrete phases: migration (autumn and spring combined), non-breeding residence phase and breeding phase. We defined the migration phase when positions were at least 200 km away from the breeding colony and still north from the southern border of the Sahara desert (latitude 15° N). All other positions were either assigned to the non-breeding residence phase in Africa or the breeding phase in Eurasia. Note that our geographical definition of the phase meant that positions could only be assigned with a certain probability to either phases based on the FLightR probability maps for each position. For example there were cases when FLightR could not decide whether a bird was either still at the breeding colony or had already departed due to uncertainty in latitude estimate. Quantitatively, FLightR might have predicted 40% probability that the positions was either still within the distance belonging to the breeding site and 60% probability the positions was further away belonging to the migration track already. To acknowledge this ambiguity, we bootstrapped 10,000 tracks by drawing a possible position from probability maps for each sun event. In the above example, this would have resulted in 4000 tracks where the bird was still at the breeding site and 6000 tracks where it had already departed. Then, we compared the ascent heights at differed phases using the data of all 10,000 tracks.

Our second hypothesis posits that twilight ascents were related to weather conditions. Testing this hypothesis required weather variables at the position of each twilight event. Since weather variables are often spatially and temporally autocorrelated, we considered the weather at the median position provided by the FLightR output as a good approximation of the weather birds might have experienced in reality (Hüppop 2003; Saino and Ambrosini 2008; Rohli and Vega 2013). To summarise the weather conditions, we included pressure at sea level, relative humidity, temperature and the modelled horizontal wind speed close to the ground as provided by the R-package RNCEP (Kemp et al. 2012) which accesses data of the NOAA database (Kalnay et al. 1996; Kanamitsu et al. 2002). These were modelled across an evenly spaced grid with a resolution of 2.5° latitude on the world coordinates and provide a forward prediction for 6 h at the time on 17 different pressure levels (Kemp et al. 2012). We only considered the data at 1000 mbar as this was the pressure level where birds flew most of the time. At each twilight event, we used the weather of 6-h forecast period which included the twilight event at the linearly interpolated median bird position. To investigate changes in weather conditions, we used the difference between the current 6-h forecast and 6-h forecast at 24 h before and at 24 h after for the variables pressure at sea level, relative humidity and temperature. We ignored change in wind because we had no clear expectation how birds could react to different aspects of wind: A change in wind direction could indicate an approaching weather situation but weather fronts will arrive in different region of the world from a different direction, and wind speed determines wind support for movement depending on the heading of the bird. Even without information on the wind, changes in the other variables are expected when large scale weather fronts are moving past the current location of the bird (Rohli and Vega 2013). We only compared stable weather versus changing weather and disregarded whether the change was negative or positive. All factors were *z*-transformed (centred on the mean and standardised by the standard deviation) allowing a comparison of their relative effect sizes.

We then investigated the dependence of twilight ascents on the changes in weather conditions. The frequency of twilight ascents was modelled as a binary variable of whether the bird had performed an ascent of at least 300-m height at each twilight event. The threshold of 300 m was chosen because about half ascents reached this altitude and because a test with a threshold of 100 m virtually gave the same result. Independent factors describing weather changes were extracted within the previous and proceeding 24 h of each of the variables pressure at sea level, relative humidity and temperature, respectively. Additional explanatory factors were the phase of the annual cycle, the type of the twilight (whether it was a sunrise or a sunset), and daily mean flight altitude (excluding the hour of twilight), which might indicate how much further a bird can ascend. To account for individuals from the same colony experiencing the same topographic and climatic conditions, we included the individual nested in the population as random factor. Twilight ascents were temporally autocorrelated indicating that ascents were clustered in time, and we account for this by using generalised linear mixed models (glmmPQL with a binomial distribution) from the package nlme, with an autoregressive process of the first order (corAR1) (Pinheiro et al. 2016). As described above, twilight events could not always be assigned unambiguously to a certain phase of the annual cycle. To solve ambiguity in the explanatory variable phase of the annual cycle, we ran the model a 100 times, and for each run, we generated new data by drawing a new track for each individual according to the probabilities provided by the FLightR output (see above). The predictions of all 100 model runs were graphically reported. Interference of the models was drawn from analysing the upper and lower 95% confidence intervals (calculated as twice the standard error) of each model (Hector 2015). Only when the value zero was not included in any of the 100 confidence intervals we considered a variable as statistically relevant.

#### Data availability statement

The datasets generated analysed during the current study are deposited in the “Movebank” repository (www.movebank.org, long-term study on migratory movement of Alpine swifts (*A. melba*) from different populations) and will become publically available after the full details on the route and timing of migration has been published. Before that they can requested from the corresponding author on reasonable request.

## Results

From the four populations, 15, 10, 3 and 6 tags had successfully recorded data for at least 11.5 months and could be used for our analysis. Seven more tags had returned but were excluded because they either ran out of battery before the season was completed (five tags) or the light sensor malfunctioned during the deployment (two tags).

**Fig. 1:**
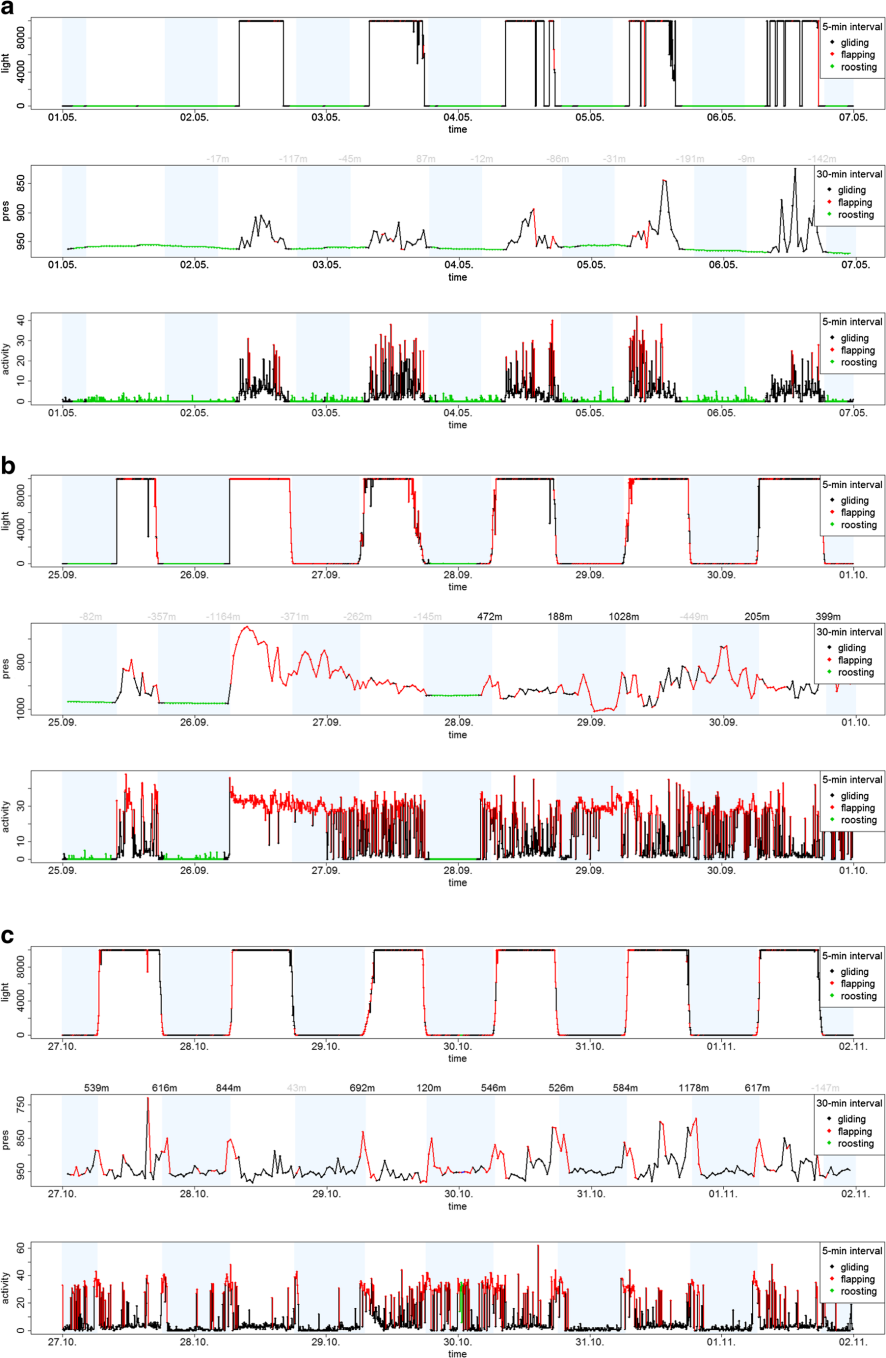
Example of raw data from a SOI-GDL3pam tag of a bird from the Swiss population during **a** the breeding phase, **b** the migration phase and **c** the non-breeding residence phase. The three panels show the light-level (every 5 min), the pressure (every 30 min) and the activity measure (every 5 min) for a period of 6 days. The grey shading area indicates night time and it was either determined using the light-level data (**b**, **c**) or it was calculated for the location of the breeding site (**a**, see text for detail). The colours of the graphs correspond to the three different flight behaviours (gliding, flapping and resting as defined in the supplement). The numbers centred above each twilight event in central panel refer to the height of the ascent at twilight relative to the mean flight altitude calculated over 6 h before and after twilight. Ascents below 300 m are shaded

The analyses of light, pressure and activity data revealed distinctive behavioural pattern during the breeding phase, migration, and the non-breeding residence phase (Fig. 1). During migration, the birds moved between consecutive twilight events by 0.17° ± 2.1° in latitude (mean and standard deviation) and 0.012° ± 1.5° in longitude. During the non-breeding residency in Africa, the averaged movements were 0.009° ± 0.46° latitude and 0.005° ± 0.31° longitude. During the breeding phase, geolocation was not possible because most twilight events were commonly classified as non-natural sun events. However, our observation at the Swiss breeding colony confirmed that birds usually attended the colony during twilight and hence, there median global movement was 0° in both latitude and longitude during this phase. Loggers then only recorded the first light when birds left the colony in the morning (Fig. 1). In contrast, during the migration and non-breeding residence phase, light data rarely recorded any shading during twilight events.

**Fig. 2:**
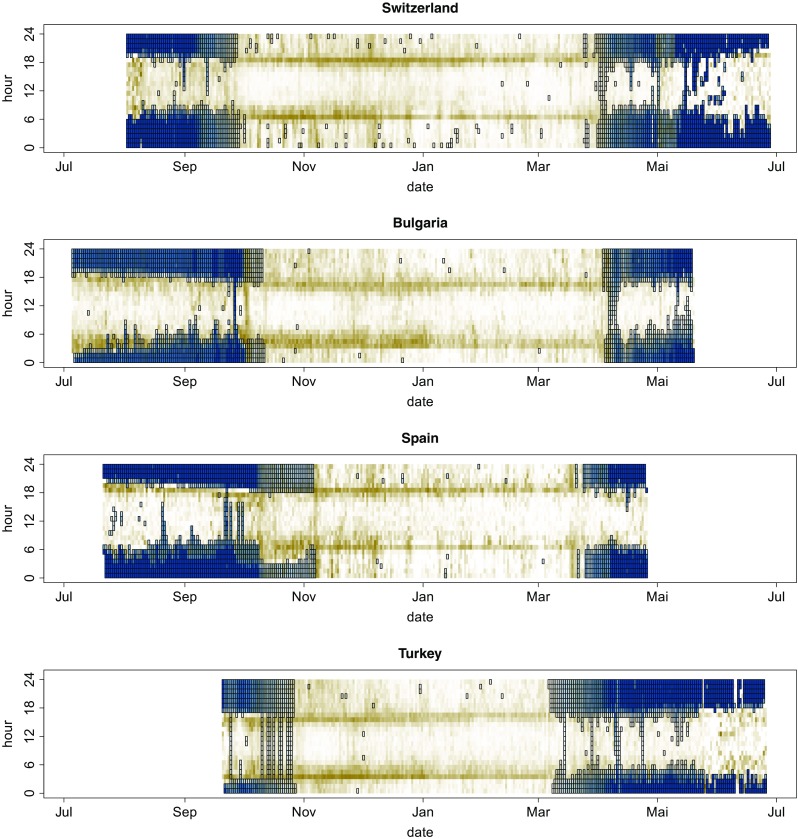
Population-specific frequency of the different flight behaviour among all individuals (gliding, flapping and resting on the ground as defined in the supplement) across the time of day (*y*-axis) and the season (*x*-axis). Each pixel summarises the frequency of the behaviour over 1 h across all individuals in the population (the number of individuals differs between days and populations). Intensity of redness of the cell represents the relative frequency (among individuals of the entire population of flapping behaviour, intensity of greenness of the cell shows frequency of resting on the ground, and white pixels show hours with predominantly gliding flight behaviour. Hours where at least one bird might have rested on the ground are framed in black. The data series started when birds were tagged at the end of the breeding season and lasted until loggers could be retrieved from the breeding birds in the previous year (capturing of birds followed different schedules at each population)

The pressure measure revealed that birds during migration sometimes flew for several hours at constantly high altitude of 2000–3000 m above sea level (ASL) (Fig. 1). The highest altitude of 5115 m ASL was recorded for a bird from the Bulgarian population during spring migration. However, the average flight altitude did not differ generally between the migration phase and the non-breeding residence phase (analysis not shown). At the non-breeding range, birds regularly reached maximal flight altitude close to the sunrise and sunset and showed varying altitudes through daylight and night time (Fig. 1). At the breeding range, birds typically reached the highest flight altitude during the day; at night, pressure levels corresponded well with local ground pressure at their colony. The best evidence to determine whether a bird spent time on the ground or in the air provided the variance in the pressure (Fig. 2, supplementary material for details).

**Fig. 3:**
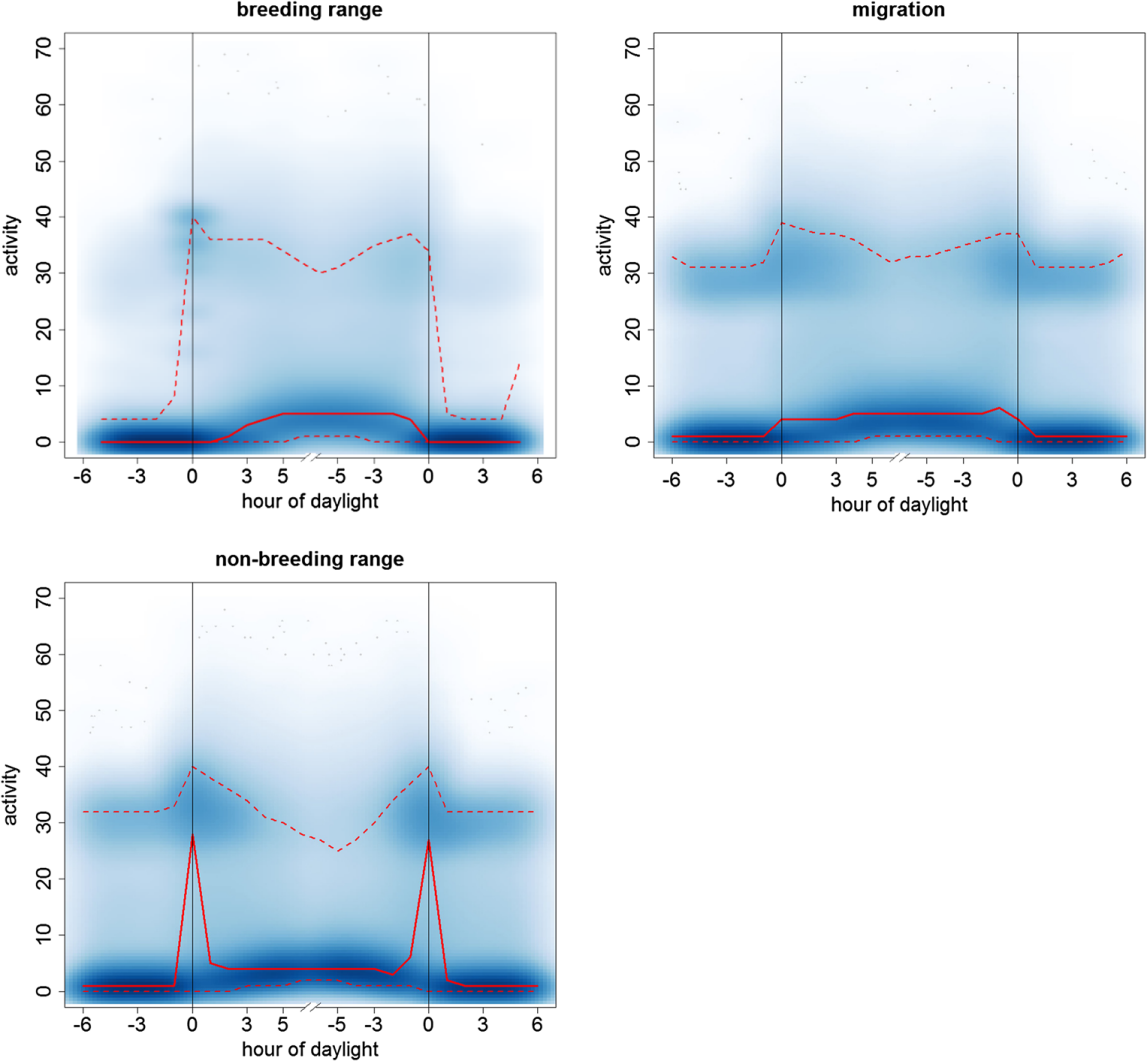
Daily course of the flight activity of alpine swifts for the three phases of the annual cycle. The blue shading shows the density distribution of the raw measure of activity (every 5 min) of each bird. The solid red line shows the median value per hour and the dotted line shows the lower and upper 95% confidence interval of the data. The hour of the twilight is marked by vertical lines, on the left hand site for sunrises and right for sunsets, and the time on the *x*-axes is shown in hours relative to the closest twilight event

Of all the air borne activity constant flapping flight was the most distinctive behaviour. The longest periods of constant flapping occurred during the migration phase (Fig. 1b during days 2 and 3, Fig. 2). Shorter periods of constant flapping also occurred on most days around the year, often cumulating around twilight (Figs. 2 and 3).

The clear peak in the activity during twilight in the non-breeding residence phase already indicated that birds had a high wing flapping frequency and therefore might have performed an ascending flight. This was confirmed by the change in flight altitude. Over 30 min, the change in altitude was positively correlated with the mean flapping activity (slope = 0.34, CI 0.33–0.35, in a mixed model with individual as random factor). However, the activity was only able to predict the change in flight altitude for 30-min intervals including a twilight event, but it was a weak predictor for all other 30-min interval during the rest of the day (slope = − 0.062, CI − 0.059 to − 0.065, in a mixed model with individual as random factor, see also supplementary material Fig. A3).

**Fig. 4:**
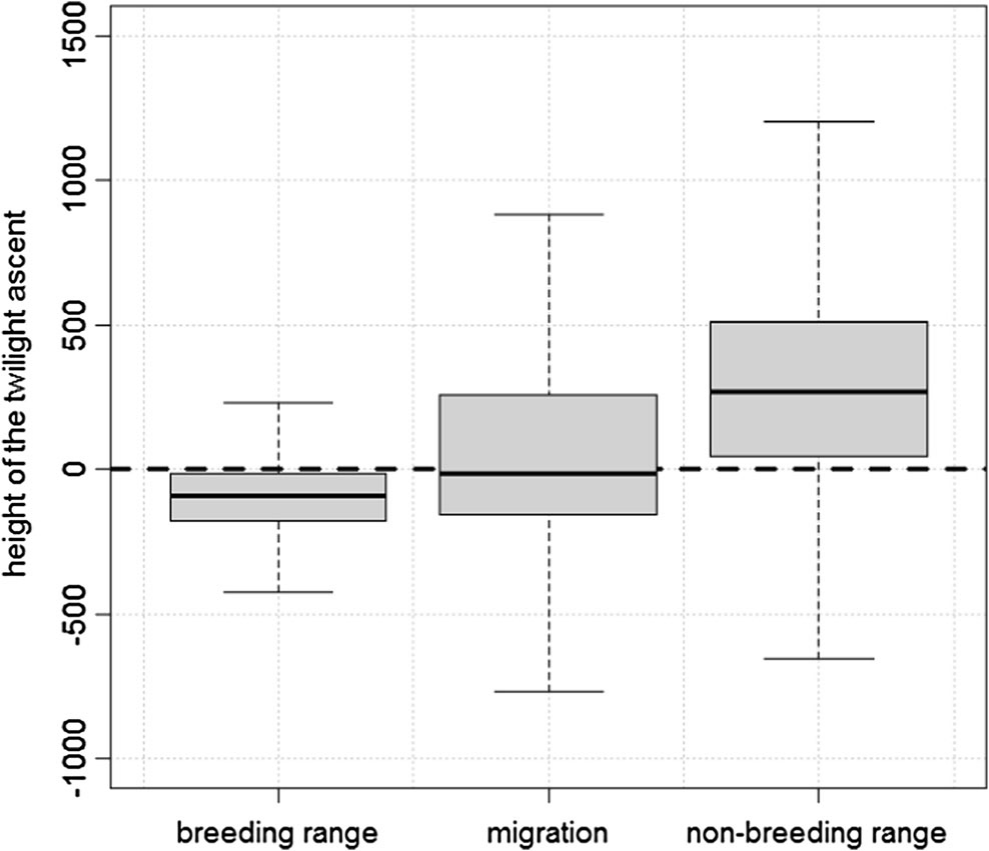
The ascent height at the twilight at the three phases of the annual cycle. The *y*-axis shows the difference between average flight altitude during twilight and the average flight for the time of 6 h around in meters. Data were resampled from the raw geolocator data according the probability of belonging to one of the three phases (see text for details). The bold dash lines marks the limit above which birds made an ascent at twilight. Below this line birds stayed at a lower altitude during twilight compared to 12-h period around the twilight. Whiskers show the upper and lower quartiles, the grey boxes contain the 50% quantile and the black lines shows the median for each phase

Only during the non-breeding residence phase flight altitudes were significantly higher around twilight compared to the rest of the day (Wilcoxon signed rank test testing median ascent height (mah) per individual mah_non-breeding_ > 0 m, *V* = 561, *p* < 0.001). All twilight ascents during this phase comprised a height difference of 266 m (42–507 m; median, upper, and lower quartiles; Fig. 4). During the migration phase the difference in the flight altitude at twilight and at the rest of the day was insignificant − 17 m (− 157–257 m; Wilcoxon signed rank test testing mah_migration_ > 0 m, *V* = 155, *p* = 0.99; Fig. 4). During the breeding phase, birds usually stay at the colony during the twilight period resulting in a much lower height at twilight of − 91 m (− 179 to − 15 m) compared to the rest of the day (Wilcoxon signed rank test testing mah_breeding_ > 0 m, *V* = 15, *p* = 1).

**Fig. 5:**
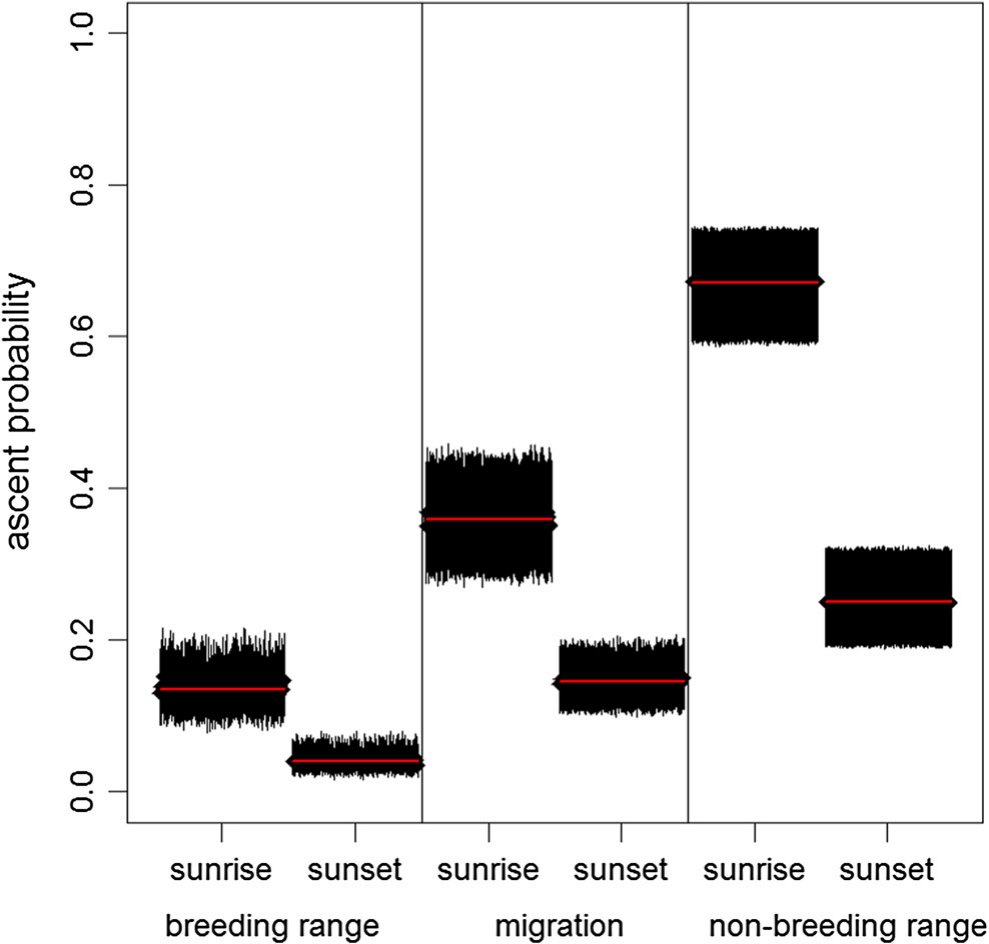
Probability of ascents in each phase of the annual cycle for sunrise and sunset. Blocks consist of estimated probabilities of hundred model runs (see text). Shown are vertical bars for the upper and lower 95% confidence interval for each model run. The red line shows the overall all mean of the estimate for each block

The model for the frequency of ascents of at least 300 m confirmed that ascents occurred with the highest frequency during the non-breeding residence phase: The difference compared to the migration phase and to the breeding phase were significant (Fig. 5). The strongest effect had the type of twilight. Ascents occurred more than twice as frequently during dawn than dusk. Both effects, the phase and the type of twilight, were robust across the 100 model runs (Fig. 5, Table A1 supplementary material). Ascents occur more frequently when birds already flew at high-altitude ASL during the 6-h period before and after the twilight, when temperature was high and wind speed was low.

**Fig. 6:**
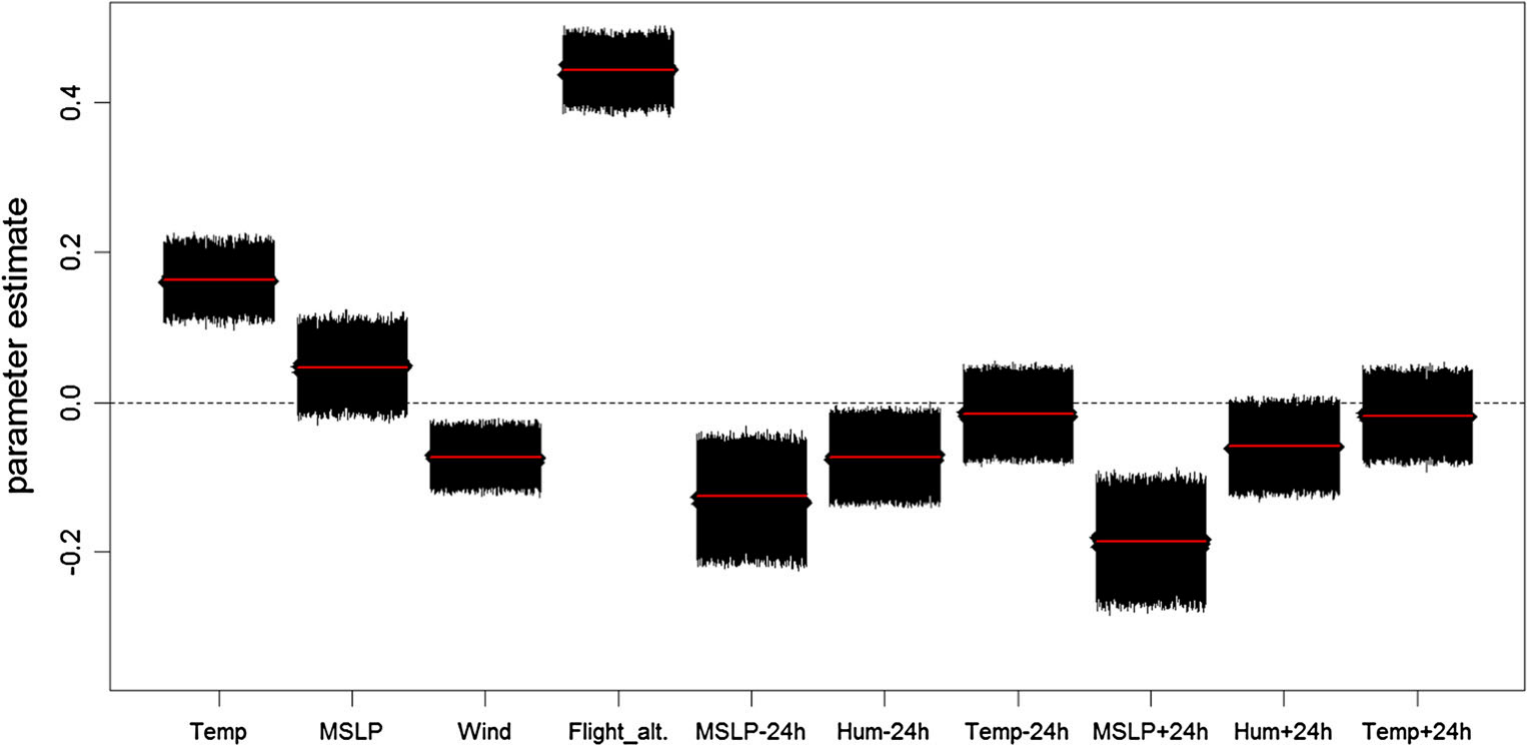
Influence of weather variables on the probability of ascents. Abbreviation of factors stand for Temp = temperature 2 m AGL, MSLP = mean sea level pressure, Wind = wind speed at the pressure level of 1000 mbar, Flight alt. = mean flight altitude of the birds for 12 h around the twilight excluding the hour of the twilight event, Hum = relative humidity of the air at the pressure level of 1000 mbar. Factors of changes in the atmosphere are labelled with suffix dependent on whether the change happened before or after the twilight event. Suffixes refers to the change that happened between the 6-h period including the twilight and the same period of the day 24 h earlier ‘−24 h’ and 24 h later ‘−24 h’. The plot shows the estimates for sunrise during migration. Blocks show the estimates and 95% confidence intervals of a hundred models with a different migratory timing for each factor in a similar way as in Fig. 5. All factors were scaled and therefore effect size can be compared between factors. Factors are significant if the bars of the CI do not include the value 0 (dotted line)

Large changes in the atmospheric pressure resulted in a decreased ascent activity of the birds, irrespective whether the change occurred in the 24 h before or after the twilight. Marginally significant was also a lower ascending probability when humidity changes occurred in the 24 h prior the twilight (some of the models had a 95% CI which include the value 0, Fig. 6). When temperature changed birds decreased ascent probability, but these effects were not significant no matter if the change happened in the 24 h before or after the twilight (Fig. 6). Birds obviously showed a higher frequency of ascents when the atmospheric conditions were stable.

## Discussion

We investigated twilight ascents as a particular aspect of flight behaviour in alpine swifts. Twilight ascents were about twice as frequent at dawn than at dusk, but the corresponding increase in flight activity was not different between the two twilight events. And they were by far most frequent during the non-breeding residence phase in Africa, compared to the breeding and migration phase. Furthermore, we can also exclude our finding is a mere artefact of a potentially bias in precision of pressure level data at different continents.

We could not find a support for the recalibration hypothesis as supposed by (Dokter et al. 2013). The frequency of ascents did not increase when Alpine swifts moved across the landscape on a global scale. Ascents were half as frequent during migration when birds cover a migratory journey of approximately 3500 km in about 2 weeks (Liechti et al. 2013) compared to stationary periods outside the breeding time when they reside at a confined site in Africa of a size as large as their breeding sites. We are faithful that the result is not an artefact of a spatially biased calculation of the ascent probability. In an unpublished pilot study, the pressure measures of the multi-sensor loggers showed a discrepancy of only 1 hPa to a nearby weather station of the Swiss meteorological institute and the NCEP data for pressure ASL which were needed to calculate the ascent height came from a global model by NOAA (Kalnay et al. 1996; Kanamitsu et al. 2002) which has been proven to be accurate by Salstein et al. (2008). Our results do not support the idea that twilight ascents represent orientation behaviour; otherwise, this behaviour should be most pronounced during migration and not during stationary periods in Africa. Most likely, Alpine swifts orient without an ascent by using celestial and magnetic cues like many other birds do (Cochran et al. 2004; Muheim et al. 2014). The alternative explanation for twilight ascents, that birds might be profiling the different strata of the atmosphere with their ascents, was also not supported by our data. Dokter et al. (2013) found evidence in common swifts for higher ascents when the height of the 280 K isocline was higher, but this relationship did not hold for any other meteorological parameters, such as wind and cloudiness. The authors therefore were not sure if birds indeed sampled the atmosphere for a temperature gradient with the ascent. In fact, other important properties could co-vary with temperature, for example, the height of convective boundary layer where upwind drafts occur, and affect the bird flight altitude independent of whether they performed a twilight ascent (Holton and Hakim 2012).

With respect to weather conditions, our results only indicate that twilight ascents occur mainly during stable pressure conditions and the frequency decreases as pressure conditions are more variable. Changing pressure is mainly associated with passing by weather fronts which bring precipitation and clouds. Instead, twilight ascents occurred mainly in calm and dry weather conditions when no synoptic weather front was moving through at the position of the bird. The frequency of ascents was also independent of the presence of clouds and our data are rather ambiguous of whether birds stayed below the limits of clouds or whether they sometimes even ascended into the clouds (see Appendix). Thus, contradictory with this hypothesis, twilight ascents did not support the idea of atmospheric profiling in relation to changes in weather conditions but is in line with much older observation that common swifts stay aloft overnight and that they select higher altitudes during warmer nights (Bruderer and Weitnauer 1972).

Because neither of the two hypotheses received convincing support, we came up with a new hypothesis, the ‘social behaviour hypothesis’, which could be tested in future studies on twilight ascents. Liechti et al. (2013) and Hedenström et al. (2016) had reported striking peaks of activity during twilight for Alpine and common swifts, respectively. Both interpreted this behaviour as diurnal ascents, although their tags did not have a pressure sensor allowing the estimation of the flight altitude. Our data include flight altitudes and show a correlation of activity peaks around twilight with ascents. Radar measurements had already shown that climb rates in swifts are correlated with the duration of wing flapping phases (Stark 1996). In our data, high peaks in twilight activity were often related to ascents. Lack (1956) described fast flight behaviour of common swifts near their breeding colony in the first and the last half an hour of the day. He called this behaviour ‘screaming parties’ (Lack 1956; Weitnauer 1980). Birds are chasing each other at very high speed, displaying flight acrobatics, intensively vocalising, and obviously engaging in social interactions with members of the same colony. A study analysing the screaming parties of common swift with video recording found a high wing beat frequency when birds attended screaming parties (Henningsson et al. 2010). Thus, peaks in activity which occur around the year at twilight could be the signature of screaming parties on our loggers and this would mean that screaming parties also occur while Alpine swifts are at high altitudes, and that they might just be beyond the detection of a ground observer. If this would be the case, the reason for twilight ascents might well be part of a social behaviour.

In fact, the timing of ascents marks an interesting parallel with social interaction of oscine birds. These birds make a ‘dawn chorus’ to attract mates and defend their territories (Catchpole and Slater 2003). Twilight ascent singing activity shows a similar daily pattern, with a major peak at sunrise and a second weaker peak at sunset. Mate attraction might be important in Alpine swifts too, as they are monogamous and colonial breeders (Arn 1960). Some oscine bird species are also known to display their singing activity during the non-breeding residence phase as it was the case with twilight ascents in Alpine swifts (Sorensen 2014; Sorensen et al. 2016).

A reason why singing occurs primarily at twilight is debated and several hypotheses were reviewed by Catchpole and Slater (2003). Time allocation of social activities competes with time allocation of foraging, and if foraging is most efficient during daylight, then social activities are expected to shift to the twilight period instead (McNamara et al. 1987). And if communication at night fails, the break in information should best be compensated with more signalling closer in time to the break with a peak especially at dawn before the next social interactions can occur (Catchpole and Slater 2003).

The latter argument actually fits better for visual communication, as acoustic communication could also work during the night and makes more sense for communication in flight. At higher altitude, birds might improve the perception of their coloration to conspecifics by ascending at twilight. With an ascent of 300 m they might see the sun about 2 min earlier than a bird flying lower. In the silhouette of a bright background sky, the light improvement might be even more pronounced.

We therefore conclude that neither the recalibration hypothesis nor the atmospheric profiling hypothesis provides a satisfyingly explanation why twilight ascent are part of such a common daily routine. The behaviour remains a mystery. We suggest that future investigations should elaborate whether the behaviour plays a role in social interaction. Evidence for such an interaction could be if ascent behaviour of breeding pairs, their offspring and other individuals of the same colony would correlate. Simultaneous ascent behaviour between individuals could indicate the protagonists involved in the interaction and this might shed light on the type of information transmitted on ascents and screaming parties. However, a comparison across the entire annual cycle would also require established information on which individuals were travelling together in time and space. Most birds in our study flew on different tracks to their non-breeding site. Future studies should concentrate on tagging breeding pairs and increase the number of individuals tagged at the same breeding colony. Augmented use of miniaturised tracking devices might hopefully accumulate the data needed to answer this question in the future.

## Electronic supplementary material

ESM 1 (DOCX 147 kb)
