# Supplementary material for: What makes Alpine swift ascend at twilight? Novel geolocators reveal year-round flight behaviour
Source: Behav Ecol Sociobiol. 2018 Feb 26;72(3):45. doi: 10.1007/s00265-017-2438-6 (PMC5847200; doi:10.1007/s00265-017-2438-6)
Supplement: Supplementary file 1 — (DOCX 147 kb) [file 265_2017_2438_MOESM1_ESM.docx]

# Supplementary material

### What makes Alpine swift ascend at twilight? Novel geolocators reveal year-round flight behaviour

### Behavioral Ecology and Sociobiology

### Authors

Christoph M. Meier^1*^, Hakan Karaardıç^2^, Raül Aymí^3^, Strahil G. Peev^4^, Erich Bächler^1^, Roger Weber^5^, Willem Witvliet^6^, Felix Liechti^1^

*Author for correspondence

Phone: +41414629923

Fax: +41414629710

E-mail: [christoph.meier@vogelwarte.ch](mailto:christoph.meier@vogelwarte.ch)

^1^ Swiss Ornithological Institute

Seerose 1

6204 Sempach

Switzerland

^2^ Elementary Science Education Department

Education Faculty

Alanya Alaaddin Keykubat University

07400 Alanya

Turkey

^3^ Catalan Ornithological Institute

Museu de Ciències Naturals de Barcelona

Pl. Leonardo da Vinci, 4-5

08019 Barcelona

Spain

^4^ Institute of Biodiversity and Ecosystem Research

Bulgarian Academy of Sciences

2, Gagarin Street

1113 Sofia

Bulgaria

^5^ Bern University of Applied Sciences Engineering and Information Technology

Jlcoweg 1

3400 Burgdorf

Switzerland

^6^ Willem Witvliet

Zuidersloot 16

1741 HL Broek op Langedijk

Netherland

Specification of the FLightR analysis

The date of sun events was identified using the threshold of 3 light units above the tag-specific minimum for darkness and ranged between 3 and 26 units for our loggers. FLightR could only deal with natural sun events including the gradual increase/decrease of light during the twilight phases. Therefore all other unnatural sun events were labelled and excluded by hand. This was done by visually checking the light curve from the minimum light value below the threshold to the maximum light value of the logger at 9984 units for a smooth slope. We restricted the analysis of FLightR to the period between the first and the last day for which tag had recorded at least two consecutive days with natural sun events. We made this assumption because previous work as shown that alpine swifts usually initiated and determined their migration with a long distance flight (Liechti et al. 2013, and see below for a further discussion of this assumption). Further, we assumed that the bird remained at the location of the breeding colony for the rest of the time as long as the geolocator was deployed. Flight altitude and flight behaviour was analysed over the entire time the geolocator was attached to the bird. For simplicity, we did not integrate the flight altitude in the calculation of the position through geolocation, although birds at a higher altitude might have experienced a sun event slightly different time than on a ground position. For example an altitude of 1000 m the sun appears four minutes earlier. Since we had already removed unnatural sun events we did not use the built in outlier filter of FLightR.

FLightR required the tag-specific information sensitive of the light-sensor to accurately identify twilight events. The information needs to be provided as the range of sun elevation angle and the range of light values on the tag which correspond to the typical light level of a sun event. We calibrated our SOI_GDL3pam tag with the parameter log.light.borders = c(2.2,7), log.irrad.borders = c(-2.7,0), and all.in$Calibration$Parameters$LogSlope[2] = 0.395 (for details see Rakhimberdiev and Saveliev 2016). FLight requires an expert guess of the movement mode of evaluated species. Swifts in general spend most of their time aloft and often fly with ground speed up to 50 km per hour (Bruderer and Boldt 2001; Henningsson et al. 2010). It is therefore possible for a swift to cover 600 km with a 12 h flight. The exact flight mode during migration in alpine swift is unknown but at least the first geolocator study found no evidence for major stop over sites between Europe and Africa (Liechti et al. 2013). We therefore chose a high probability of movement between sun events of 0.25, an average movement step size between twilight of 600 km with a large, uninformative standard deviation of 300 km. The maximum movement step size was restricted to 2000 km. The particle filter of FlightR was run with one million particle and tracks were stipulated to return to breeding colony since all tag were running until the date of recapture.

Behaviour categories

We used pitch, activity and standard deviation in pressure to assign each 5-min time interval to one of three behaviour categories. When birds were resting on the ground the pressure-values on the tag were only affected by slow atmospheric changes in pressure. We calculated for pressures measure the standard deviation with the two proceeding and following pressure measures. The distribution of these standard deviations showed a clear bimodal distribution allowing a clear separation between resting on the ground and flying behaviour (Fig. A1). The threshold for variance in the pressure was done by eye. We tested several values and evaluated them against a short independent data set of a bird for which we had recorded data activity at a constant rate with 10 hHz. Based on this test we chose a threshold which showed the lowest false classification of either roosting or flying behaviour.


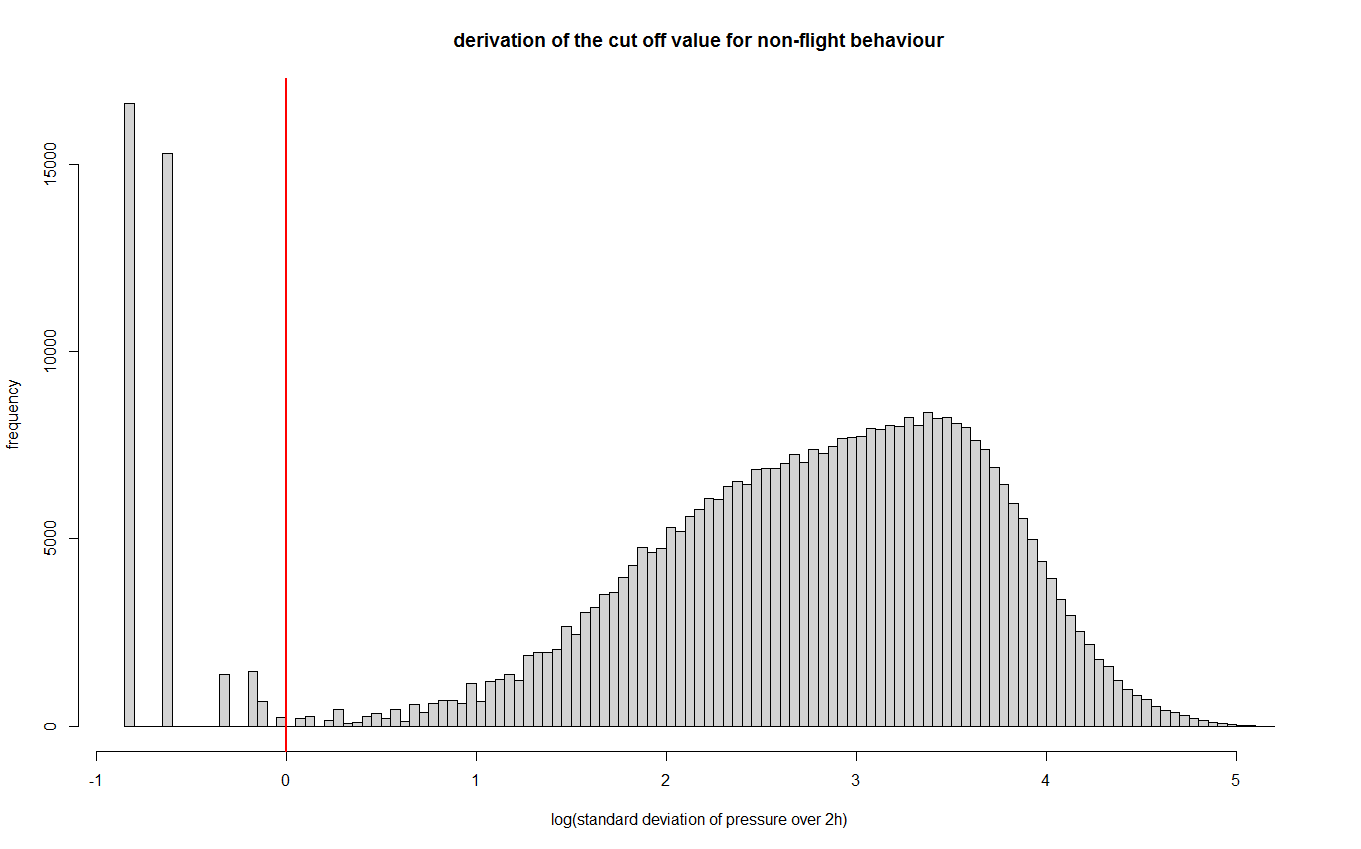


**Fig. A1** Histogram of the standard deviation of the pressure over the 2.5 hour interval. Values showed a bimodal distribution on a logarithmic scale. The red line shows the separation between resting behaviour on the ground and flying behaviour

Similarly, the activity measures at each 5 minutes interval showed a bimodal distribution with a saddle point at an activity-level of 21. Behaviour below this threshold we called “gliding” and behaviour above the threshold “flapping”. For flapping the intensity of activity was positively correlated with ascending flight behaviour, whereas for gliding we found no such relationship (Fig. A2). The threshold of activity was simply chosen at the settle point of the bimodal distribution histogram of activity levels.

The only result of the paper which depends on this classification is shown in figure 2. We concluded from this result that birds indeed remained air borne for the entire season. Changing the threshold for variance in pressure towards positive values would not change much as there are very few small positive values. Changing the values towards a negative threshold would reduce the green cells in figure 2 and strengthen our conclusion that birds where not roosting during the non-breeding phase.


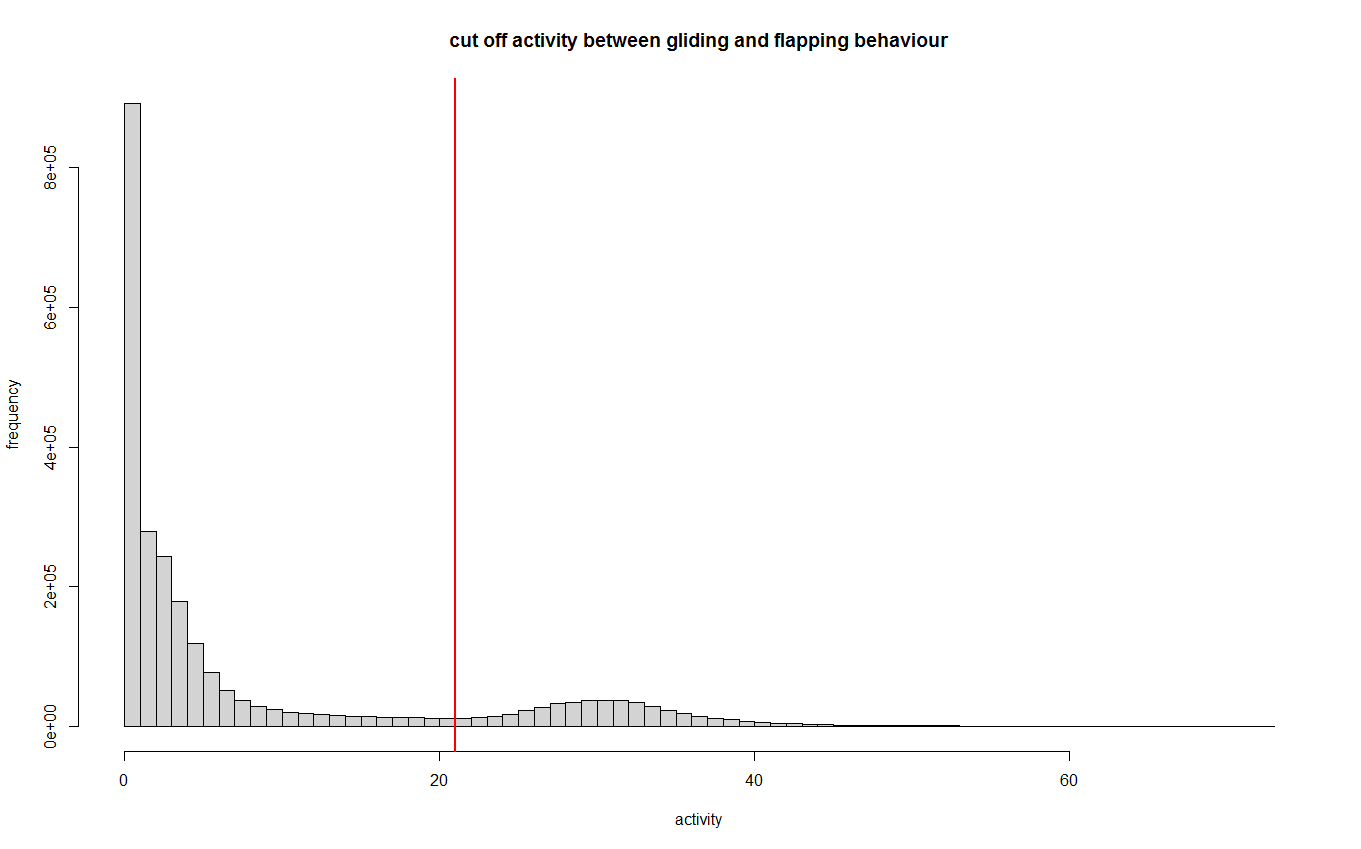


**Fig. A2** The distribution of raw activity values every 5 minutes. The red line shows the separation between gliding and flapping behaviour

**
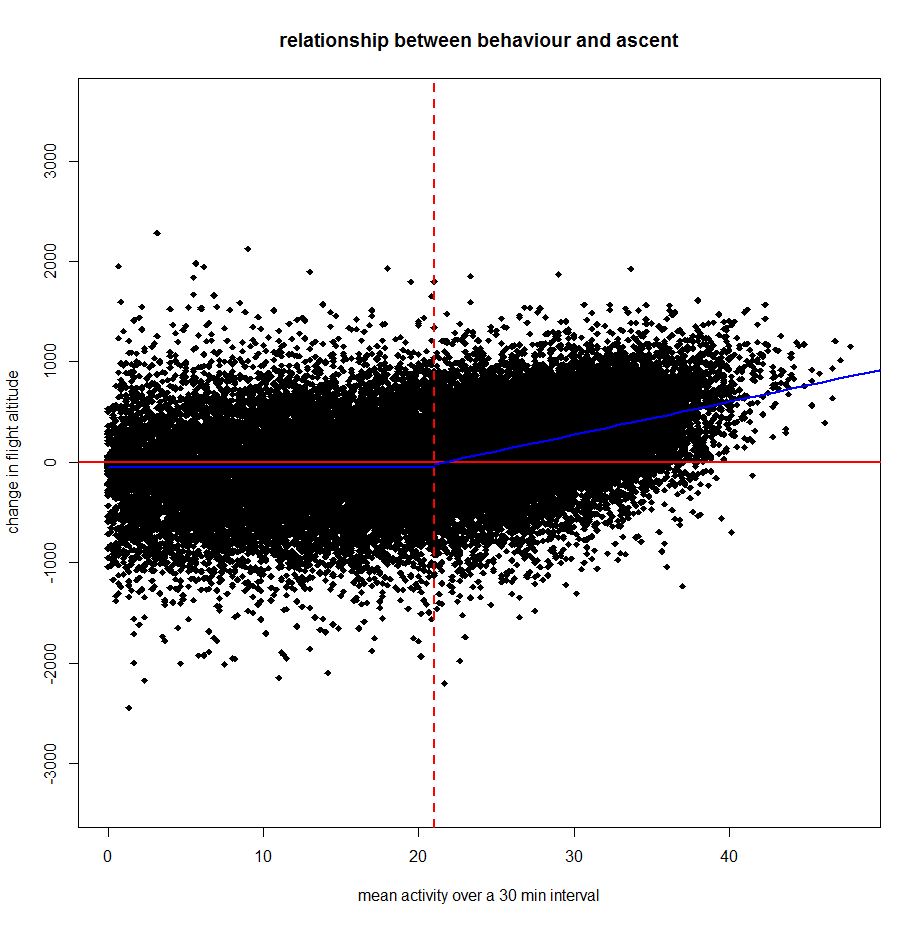
Fig. A3** Relationship between the activity measure and the change in flight altitude for all 30 minutes intervals including a twilight event. Activity measures, on the x-axes, were averaged over 30 minutes to match with the difference of two consecutive measures of flight altitude, on the y-axes, over the same period. Activity values below 21 units (dashed red line) indicate gliding behaviour and higher values correspond to flapping behaviour. The solid lines shows the linear relationship between activity and change in flight altitude across the whole range of activity measures (in red) and separate across the range for gliding and flapping behaviour (Liechti et al. 2013).

We tested this relationship with a linear mixed model with a gaussian error distribution. Change in flight altitude was the only fixed effect and individum as random effect. We used the R-package arm to draw interference for the model on the slope (Korner-Nievergelt 2015; Gelman and Su 2016). The slope for mean activity was -0.062 CI -0.059 - -0.065 for values below 21 units and 0.34 CI 0.33 - 0.35 for values equal or greater 21 units.

Another way to test the hypothesis of whether twilight ascents served the birds to update their internal map for navigation is to see whether ascents correlated with day by day movement recorded by the geolocator. Either birds might ascend to collect information prior to starting the journey, thus an ascent should occur more frequently before they make a long distance movement, or they might ascend just after they have arrived from a long distance movement because they needed to re-orienteer at the new site. We tested both option and found that in neither case the ascents of at least 300 m were associated with long distance moves. Thus, the result could not add evidence that twilight ascents might be important for orientation.


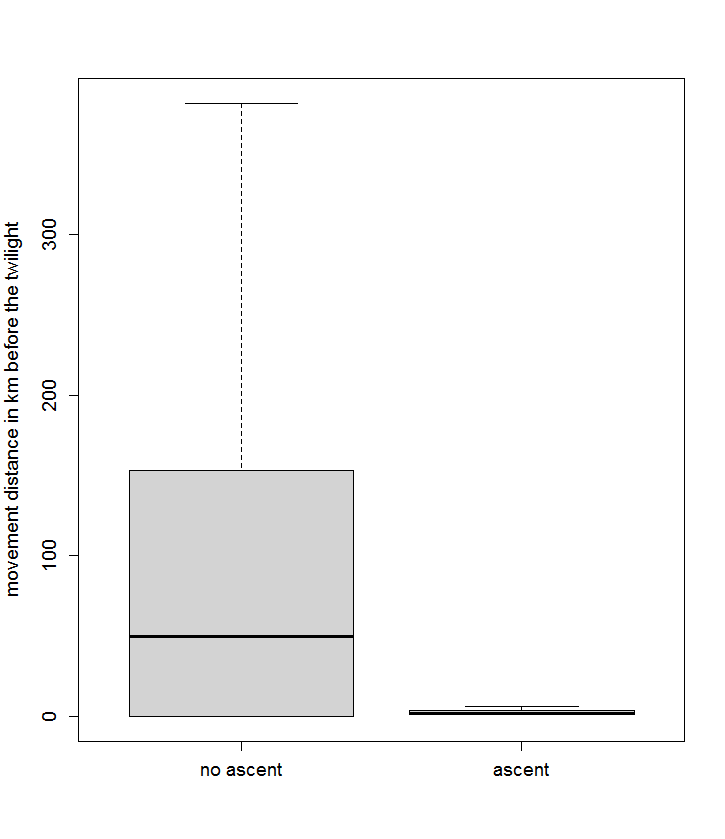

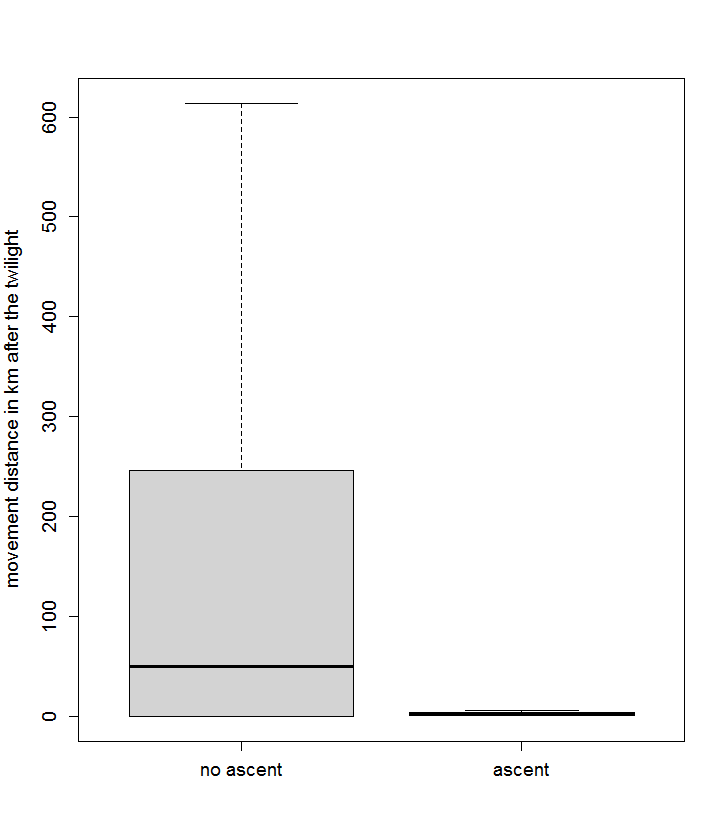


**Fig. A4** The figure shows the movement distance before (left panel) and after (right panel) a twilight dependent on whether the bird made an ascent of at least 300 m at the twilight

We investigated if clouds had influence on the motivation of birds to perform an ascent during twilight. The presence of clouds could influence the motivation in three ways. Firstly, clouds might block the few on land marks and might prevent birds from ascending, secondly, clouds might be the reason for birds to ascent because they need to escape the misty visibility on the ground, and thirdly clouds might indicate the instability in the atmosphere and indicate condition at which birds might want to profile the structure of the atmosphere with an ascent. We expected birds to avoid ascending into the cloud in the first case, birds to rise above the clouds in the second case and birds to ascend with a higher frequency in the presence of clouds in the third case. We analysed data on clouds in the lowest atmospheric layer where birds actually stay (up to 625 hPa which corresponds to approximately 4000 m *ASL*). We calculated the ratio of ascents with and without clouds at each stage and we analysed the altitude of ascending birds relative to the lower and the upper bottom of the cloud. We found none of the three hypotheses to be fulfilled (Table A1, A2). The motivation of ascents was only dependent on the annual phase rather than the occurrence of clouds. Birds ascended rarely above the clouds, but ascents into the clouds were also observed in about a third of all cases. Therefore, it seems unlikely that birds ascended neither for the overview in the landscape nor for inspecting the instability of the atmosphere. It is, however, difficult to make further conclusion from the cloud data. The information on cloud layers was collected with radar and lidar systems from satellites and interpolated in space. The data therefore is only of low spatial and temporal resolution and might provide only limited information on local cloud coverage.

Table A1. The table shows the frequency of ascending at each stage and dependent on cloud cover in the lower atmosphere (<625 hPa). The lowest row shows the frequency of clouds at the time of twilight for each stage.

**Table A1** Frequency of clouds in each of the three annual phases

|  | Breeding | migration | Non-breeding |
| --- | --- | --- | --- |
| Clouds | 0.03 | 0.13 | 0.47 |
| No clouds | 0.05 | 0.15 | 0.46 |
| Cloud freq. | 0.76 | 0.57 | 0.88 |

**Table A2** The table shows the position of the birds relative to the limits of the cloud at each stage

|  | Breeding | migration | Non-breeding |
| --- | --- | --- | --- |
| Above | 0.00 | 0.03 | 0.00 |
| inside | 0.34 | 0.26 | 0.30 |
| Below | 0.66 | 0.71 | 0.70 |

**References**

Bruderer B, Boldt A (2001) Flight characteristics of birds. Ibis 143:178–204

Gelman A, Su Y-S (2016) arm: Data Analysis Using Regression and Multilevel/Hierarchical Models, https://CRAN.R-project.org/package=arm

Henningsson P, Johansson LC, Hedenström A (2010) How swift are swifts *Apus apus*? J Avian Biol 41:94–98

Korner-Nievergelt F (2015) Bayesian data analysis in ecology using linear models with R, BUGS, and Stan. Academic Press an imprint of Elsevier, Amsterdam Boston

Liechti F, Witvliet W, Weber R, Bächler E (2013) First evidence of a 200-day non-stop flight in a bird. Nat Commun 4:2554

Rakhimberdiev E, Saveliev A (2016) FLightR: SSM for solar geolocation, https://github.com/eldarrak/FLightR
